# Differential effects of diesel exhaust particles on T cell differentiation and autoimmune disease

**DOI:** 10.1186/s12989-018-0271-3

**Published:** 2018-08-24

**Authors:** Chelsea A. O’Driscoll, Leah A. Owens, Madeline E. Gallo, Erica J. Hoffmann, Amin Afrazi, Mei Han, John H. Fechner, James J. Schauer, Christopher A. Bradfield, Joshua D. Mezrich

**Affiliations:** 1 Department of Surgery, Division of Transplantation, School of Medicine and Public Health, University of Wisconsin-Madison, 600 Highland Avenue MC7375, Madison, WI 53792 USA; 2 Molecular and Environmental Toxicology Center, School of Medicine and Public Health, University of Wisconsin-Madison, Madison, WI USA; 3 Molecular and Applied Nutrition Training Program, College of Agricultural and Life Sciences, University of Wisconsin-Madison, Madison, WI USA; 4 Wisconsin State Laboratory of Hygiene, Madison, WI USA; 5 Civil and Environmental Engineering, College of Engineering, University of Wisconsin-Madison, Madison, WI USA; 6 McArdle Laboratory for Cancer Research, School of Medicine and Public Health, University of Wisconsin-Madison, Madison, WI USA

**Keywords:** Diesel exhaust particles, Particulate matter, T cell differentiation, Autoimmune disease, Aryl hydrocarbon receptor, Metabolism, Cytochrome P450

## Abstract

**Background:**

Exposure to particulate matter (PM) has been associated with increased incidence and severity of autoimmune disease. Diesel PM is primarily composed of an elemental carbon core and adsorbed organic compounds such as polycyclic aromatic hydrocarbons (PAHs) and contributes up to 40% of atmospheric PM. The organic fraction (OF) of PM excludes all metals and inorganics and retains most organic compounds, such as PAHs. Both PM and OF increase inflammation in vitro and aggravate autoimmune disease in humans. PAHs are known aryl hydrocarbon receptor (AHR) ligands. The AHR modulates T cell differentiation and effector function in vitro and in experimental autoimmune encephalomyelitis (EAE), a murine model of autoimmune disease. This study aims to identify whether the total mass or active components of PM are responsible for activating pathways associated with exposure to PM and autoimmune disease. This study tests the hypothesis that active components present in diesel PM and their OF enhance effector T cell differentiation and aggravate autoimmune disease.

**Results:**

Two different diesel samples, each characterized for their components, were tested for their effects on autoimmunity. Both diesel PM enhanced effector T cell differentiation in an AHR-dose-dependent manner and suppressed regulatory T cell differentiation in vitro. Both diesel PM aggravated EAE in vivo. Fractionated diesel OFs exhibited the same effects as PM in vitro, but unlike PM, only one diesel OF aggravated EAE. Additionally, both synthetic PAH mixtures that represent specific PAHs found in the two diesel PM samples enhanced Th17 differentiation, however one lost this effect after metabolism and only one required the AHR.

**Conclusions:**

These findings suggest that active components of PM and not total mass are driving T cell responses in vitro, but in vivo the PM matrix and complex mixtures adsorbed to the particles, not just the OF, are contributing to the observed EAE effects. This implies that examining OF alone may not be sufficient in vivo. These data further suggest that bioavailability and metabolism of organics, especially PAHs, may have an important role in vivo.

**Electronic supplementary material:**

The online version of this article (10.1186/s12989-018-0271-3) contains supplementary material, which is available to authorized users.

## Background

Air pollution has become a leading risk factor for both disease and death worldwide. Increases in global air pollution have occurred concurrently with a dramatic increase in autoimmune incidence [1–4]. There are more than 80 autoimmune diseases currently recognized and worldwide prevalence is increasing by over 12.5% each year [3, 5]. While genetic predispositions play a role in incidence of autoimmune disease, recent epidemiologic studies suggest a multifactorial etiology [6, 7]. Exposure to air pollution, specifically particulate matter (PM), is associated with aggravation of various autoimmune diseases including type 1 diabetes, multiple sclerosis (MS), rheumatoid arthritis, and systemic lupus erythematosus [8–24]. Despite the strong association between exposure to PM and autoimmunity, little is known about the physiochemical properties of PM responsible for aggravating autoimmune disease.

Diesel PM is a complex mixture composed of an elemental carbon core and adsorbed organic compounds as well as small amounts of sulfates, nitrates, metals, and other trace elements [25]. These particles have a large surface area which makes them an excellent medium for adsorbing organics [25]. The organic compounds in diesel PM, including polycyclic aromatic hydrocarbons (PAHs), average about 20–40% of total mass of diesel PM [25, 26]. The PAHs account for approximately 1% of the total mass of the diesel particle, but they are potential disease causing and cancer-causing agents [25, 27]. In addition, diesel PM makes up approximately 6% of ambient atmospheric PM in the United States and this may be as high as 36% in urban areas [25]. Diesel PM is emitted directly from diesel-powered engines (primary source) and can be formed from gaseous PM precursors (secondary source) that undergo chemical reactions in the atmosphere [25]. Atmospheric PM is a complex mixture formed from a combination of primary sources that emit PM directly into the atmosphere and secondary sources that emit gaseous PM precursors that oxidize in the atmosphere to form PM [28, 29]. Similarly, some PM components, like diesel PM, are emitted directly into the atmosphere, and other PM components are formed by chemical reactions of these precursors [29]. The diverse primary emission sources and secondary chemical reactions that generate atmospheric PM components lead to complex mixtures of PM components that include metals, nitrates, sulfates and diverse organic compounds like PAHs [30, 31]. This complexity of real world PM makes pinpointing the pathologically significant components of PM difficult to elucidate.

Attempts have been made to identify the active component(s) of PM responsible for aggravating disease by testing the effects of the intact PM or the chemically-extracted organic fraction (OF) adsorbed to it [32]. All metals and inorganics are excluded during OF extraction and most organic compounds, including most toxicants such as PAHs, are retained. Both PM and the OF have been shown to increase reactive oxygen species, incite inflammation in vitro, and are associated with cancer, respiratory, cardiovascular, and autoimmune disease incidence in humans [32–35]. Inhaled PM, especially organics, contain a soluble fraction that, readily dissolved in biological fluids, can undergo metabolic transformations after deposition in the lung [36]. These reactions along with the PM matrix, total composition, and interactions between constituents likely all contribute to disease outcomes.

The chemically-extracted OF of PM has been shown to exhibit genotoxic effects and constitute a public health hazard [37]. This fraction of PM constitutes up to 40% of total mass of diesel PM [25, 26] and is composed of various organic constituents, including PAHs, among which 16 are classified by the U.S. EPA as priority pollutants [25, 27]. PAHs present in the OF of PM are disease and cancer causing agents [25, 27] and known ligands of the aryl hydrocarbon receptor (AHR) [38]. The AHR is a ligand activated transcription factor that responds to both endogenous and exogenous ligands and activates cytochrome P450 (CYP) metabolizing enzymes among other targets [39]. The AHR has been shown to modulate T-cell differentiation and effector functions in vitro and in experimental autoimmune encephalomyelitis (EAE), a murine model of MS [40, 41]. EAE is a model of MS in which the myelin oligodendrocyte (MOG_35–55_) peptide is injected subcutaneously with adjuvant to activate the innate and adaptive immune responses leading to demyelination and inflammatory infiltration in the central nervous system (CNS) [42, 43]. These animals essentially lose tolerance to an endogenous protein in the CNS [42, 43]. Our recent work has found that an ambient urban dust particulate, SRM1649b, enhances T helper (Th)17 differentiation [44]. Th17 as well as Th1 cells play a central role in the pathology of many autoimmune diseases [45–50]. These results suggest PM may aggravate autoimmune disease via AHR-mediated augmentation of effector T cell responses.

The current study addresses the role of PM exposure and autoimmune disease by investigating whether the total mass of PM or its active components are responsible for activating pathways associated with autoimmune disease in the context of studies that show linkages between exposure to PM and autoimmune disease. By determining the differential effects of the organic fraction of PM compared to total PM on regulatory and effector T cell differentiation in vitro and on disease severity in a murine model of MS, this study tests the hypothesis that active components present in diesel exhaust particles (DEPs) and their OFs enhance effector T cell differentiation and aggravate autoimmunity. More specifically, PAHs present in DEPs and their OFs enhance effector T cell differentiation via the AHR and lead to worsened autoimmunity. Cumulatively, the findings of this study suggest that active components of PM are the primary driving factor in T cell responses in vitro. However, in vivo the PM matrix and complex composition of the mixtures adsorbed to PM, not just the OF, are responsible for the observed effects on autoimmune EAE. This implies that examining OF extracts alone may not be sufficient in vivo*,* and findings relevant to one sample of PM pollution may not apply to a different source or mixture*.* These data further suggest that the bioavailability and metabolism of organics, specifically PAHs, maintain crucial roles in in vivo responses that may not be predicted in vitro. Overall, we have found that even PM derived from similar sources do not have similar impacts on autoimmune disease and that bioavailability and metabolism of organics play a role in vivo. These data hold important implications on the regulation of PM sources to reduce the impacts of PM pollution on autoimmune disease.

## Methods

### Mice

Wild-type (WT), C57BL/6 J mice were obtained from Jackson Laboratories (stock# 000664) or bred in house in a Specific Pathogen Free facility. Christopher Bradfield provided *Ahr* null (*Ahr*^−/−^) [51] and *Cyp1a1*^−/-^*Cyp1b1*^−/-^*Cyp1a2*^−/−^ (*CypTKO*) mice on a C57BL/6 J background. All these genotypes have been backcrossed into the C57BL/6 J background for eight generations, ensuring that the knockout genotypes reside in a genetic background that is > 99.8% C57BL/6 J [52]. All mice were maintained under specific, pathogen free conditions. All animal experiments were performed in accordance with protocols approved by the School of Medicine and Public Health (SMPH) Institutional Animal Care and Use Committee at the University of Wisconsin-Madison.

### Particulate matter (PM) sample preparation

The PM standard reference materials (SRMs) 1650b and 2975 were obtained from the National Institute of Standards and Technology (NIST) (Gaithersburg, MD). Dispersed suspensions of SRM1650b and SRM2975 were created by sonication in sterile phosphate buffered saline (PBS) for 45 min in a cooling water bath. Samples were tested for endotoxin using Pierce LAL Chromogenic Endotoxin Quantification Kit (Thermo Scientific) per manufacturer’s instructions. Two different dilutions were tested from a stock of 12.5 mg/mL, 1:10 and 1:1000 (Additional file 1: Figure S1B). The concentration used at the highest dose in in vitro experiments was 1:1000 or 12.5 μg/mL. Both SRM1650b PM and SRM2975 PM endotoxin levels were very low, < 0.08EU/mL (Additional file 1: Figure S1B).

### Organic fraction (OF) extraction

The OF extractions were all prepared by the Wisconsin State Lab of Hygiene (WSLH) (Madison, WI). The organic fraction of the diesel PM samples was isolated by extracting the samples with methylene chloride (DCM) using a Soxhlet extractor. PM samples that were collected as powders (i.e. SRMs) were placed in cellulose thimbles to contain the sample. The methylene chloride extraction was conducted for 24 h with nominally 5 solvent cycles per hour. The extracts were then reduced in volume by evaporating the solvent by directing a high-purity, gaseous nitrogen stream into the sample, which was held at 50 °C in a water bath. The solvent evaporation continued until the extract volume reached 0.1 mL. 1.0 mL of hexane was then added to the concentrated extract and the volume was reduced by the nitrogen blow down to 0.1 mL. This process was repeated twice to transfer the extract into hexane and remove the methylene chloride. After the three hexane transfers, 0.2 ml of dimethyl sulfoxide (DMSO) was added the residual hexane was evaporated with nitrogen under the same conditions for 30 min. Additional DMSO was then added to obtain the final extract concentration. PAH levels were measured in the extracts using a GC/MS (6980 GC and a 5973 Inert MS; Agilent) (Additional file 1: Table S1).

### Preparation of PAH mixtures matching diesel exhaust particles

Representative PAH mixtures of SRM1650b and SRM2975 were made by creating synthetic mixtures of 15 standard PAHs in similar concentrations as found in SRM1650b and SRM2975. The PAH mixtures in this study are synthetic mixtures that replicate the milieu of 15 PAHs present in DEPs. These PAHs were chosen as they are on the Environmental Protection Agency’s (EPA) list for concern [53]. Individual PAHs were purchased from AccuStandard (Z-013-SET). All samples were prepared by the Wisconsin State Lab of Hygiene (Madison, WI). The PAH standards were transferred into a centrifuge tube and the solvent (DCM or MeOH) was blown down under nitrogen in a 50 °C water bath until 0.1 mL remained. Approximately 2 mL of hexane was added and blown down under nitrogen in a 50 °C water bath until 0.1 mL remained. This process was repeated three total times to ensure all the DCM was gone and the final solution was in hexane only. The appropriate amount of DMSO was added and the tube was placed in the 50 °C water bath under nitrogen until all hexane was removed and the PAHs were left in DMSO. PAH concentrations were based on PAH levels present in the samples at the highest dose, 10 μg/mL OC. PAH levels were measured in the mixtures using a GC/MS (6980 GC and a 5973 Inert MS; Agilent) (Additional file 1: Table S2).

### Experimental autoimmune encephalomyelitis (EAE)

#### PM treatment

**Fig. 1:**
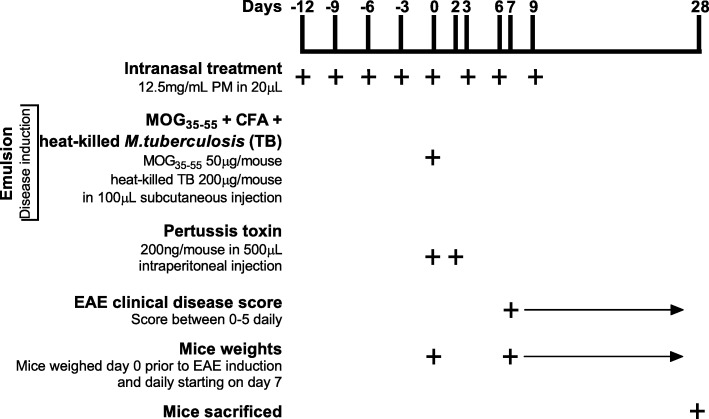
EAE Methods. Starting on day − 12 mice every 3 days until day 9, age-matched female mice receive intranasal treatment of SRM1650b PM or OF, SRM2975 PM or OF, and corresponding solvent or PBS control. The mice received 12.5 mg/mL PM or 250 μg PM in 20uL for each dose. On day 0, prior to injections, mice were weighed. A subcutaneous injection of 100 μL MOG_35–55_ emulsion was given between the shoulder blades of each mouse and an intraperitoneal injection of 200 ng/mouse pertussis toxin in 500 μL was also given. The emulsion contained 50 μg/mouse MOG_35–55_ in CFA and 200 μg/mouse heat-killed *M. tuberculosis*. Additionally, on day 2 mice received 200 ng/mouse pertussis toxin in 500 μL intraperitoneally. Mice were scored for clinical symptoms and weighed daily starting on day 7 until day 28 when the mice were sacrificed. Abbreviations: EAE, experimental autoimmune encephalomyelitis; SRM, standard reference materials; PM, particulate matter; OF, organic fraction; PBS, phosphate buffered saline; MOG, myelin oligodendrocyte glycoprotein

Age-matched (approximately 9-week-old) C57BL/6 J WT females were exposed intranasally to SRM1650b PM, SRM2975 PM, or PBS control. Female mice are used because they are more susceptible to EAE than male mice [43, 54]. The mice were exposed to 12.5 mg/mL PM or 250 μg PM of SRM1650b, SRM2975 PM, and PBS control in a total of 20 μL per dose. The 12.5 mg/mL PM dose was chosen as this was the maximum PM that could be sonicated and suspended in PBS. The mice were dosed with 20 μL of treatment or control intranasally 8 times starting at day − 12 every 3 days until day 9 after induction (Fig. 1). Mice were anesthetized via inhalation with isoflurane for each intranasal treatment.

#### OF treatment

Age-matched (approximately 9-week-old) C57BL/6 J WT females were exposed intranasally to SRM1650b OF, SRM2975 OF, or solvent control all diluted in PBS. Mice were exposed to 12.5 mg/mL SRM1650b OF, SRM2975 OF, and solvent control all diluted into PBS. SRM1650b and SRM2975 OF were used at 12.5 mg/mL or 250 μg PM in a total of 20 μL per dose. The mice were dosed with 20 μL of treatment or control intranasally 8 times starting at day − 12 every 3 days until day 9 after induction (Fig. 1). Mice were anesthetized via inhalation with isoflurane for each intranasal treatment.

#### EAE disease induction and course

Disease was induced on day 0 by injecting a myelin oligodendrocyte glycoprotein (MOG_35–55_) emulsion and pertussis toxin (Fig. 1). First, 500 μL of pertussis toxin was injected intraperitoneally (IP) at 200 ng per mouse. Next, 100 μL of the MOG_35–55_ emulsion was injected subcutaneously between the shoulder blades of each mouse. For the emulsion, 50 mg MOG_35–55_ peptide (Tocris) was prepared and mixed in Complete Freund’s Adjuvant (CFA) (BD) augmented with 4 mg/mL heat-killed *M. tuberculosis* (Difco) at a 1:1 ratio. The heat-killed *M. tuberculosis* was present in the emulsion at 200 μg/mouse and MOG_35–55_ peptide was present in the emulsion at 50 μg/mouse. Mice were anesthetized with isoflurane and weighed prior to injection on day 0.

Additionally, on day 2, or 44–52 h after the initial pertussis injection, the mice were injected with another 500 μL of pertussis toxin IP as a booster (Fig. 1). Mice were scored and weighed daily starting day 7 after induction and sacrificed on day 28 (Fig. 1). The mice were scored according to a standard procedure as follows: 0, no clinical symptoms; 0.5, partially limp/flaccid tail, 1, limp/flaccid tail; 2, hind limb weakness with incomplete paralysis, loss in coordinated movement, hind impaired righting reflex; 3, partial paralysis of hind limbs; 4, complete hind limb paralysis and 5, paraplegia or moribund. Mice were monitored and managed for pain and discomfort.

Mice were analyzed solely for clinical endpoints. Peak score was calculated as the highest clinical score mice achieved during the experiment. Cumulative score is the sum of all the clinical scores throughout the experiment duration. Mice that died due to disease were given a score of 5 and that score was carried until the end of the experiment. Day of onset represents the first day after EAE induction that a score greater than 0 is noted.

#### In vivo dosing relevance

The mice were dosed with 20 μL of 12.5 mg/mL PM of SRM1650b OF and SRM2975 OF, and Table 1 shows the nanograms of each PAH per exposure for the OF (Table 1). The 15 PAHs in Table 1 and Table 2 are the same 15 PAHs chosen from the EPA priority pollutant list and included in the synthetic DEP PAH mixtures. The 8 h and 24 h PAH air exposures are equivalents that were calculated based on the mass of PAHs in the 20 μL dose and amount of air a mouse would breathe in 8 h and 24 h (Table 1) [55]. Additionally, the nanograms of each PAH in a 20 μL exposure of 12.5 mg/mL SRM1650b PM or SRM2975 PM were calculated for the PM exposure (Table 2). The table shows the nanograms of each PAH in a 20 μL exposure and calculated 8 h and 24 h air exposure equivalents (Table 2) [55]. The calculated amounts for the amount of air a mouse would breathe were 0.01m^3^ for 8 h and 0.03m^3^ for 24 h. The permissible exposure level of PAHs set for humans by Occupational Safety and Health Administration (OSHA) is 0.2 mg/m^3^ (200,000 ng/m^3^) which is an 8-h time-weighted average permissible exposure limit [56]. This demonstrates that the OF and PM in vivo exposure the mice received is within the OSHA standard for humans and can be considered a relevant exposure.

**Table 1:** In vivo OF Exposure of PAHs

PAHs	ng PAH per in vivo OF exposure			ng PAH per in vivo OF exposure		
	SRM1650b	8 h	24 h	SRM2975	8 h	24 h
Phenanthrene	218.86	21,886	7295.22	50.583	5058.3	1686.1
Anthracene	13.790	1379.0	459.67	0	0	0
Fluoranthene	231.36	23,136	7711.89	115.13	11,513	3837.5
Pyrene	212.25	21,225	7075.05	0	0	0
Benz[a]anthracene	49.024	4902.4	1634.13	0	0	0
Chrysene	193.61	19,361	6453.59	29.519	2951.9	983.97
Benzo[b]fluoranthene	38.278	3827.8	1275.92	38.830	3883.0	1294.3
Benzo[k]fluoranthene	23.077	2307.7	769.23	0	0	0
Benzo[e]pyrene	30.440	3044.0	1014.65	3.2600	326.00	108.67
Benzo[a]pyrene	6.3570	635.70	211.90	0	0	0
Perylene	0	0	0	0	0	0
Indeno[1,2,3-cd]pyrene	27.830	2783.0	927.65	4.9558	495.58	165.19
Benzo[g,h,i]perylene	31.017	3101.7	1033.9	0	0	0
Dibenz[a,h]anthracene	0	0	0	0	0	0
Picene	0	0	0	0	0	0
Sum of PAHs	1075.9 ng	107,590 ng/m^3^	35,863 ng/m^3^	242.27 ng	24,227 ng/m^3^	8075.8 ng/m^3^

The nanograms of each PAH in the 20 μL OF exposure were calculated based on a dose of 12.5 mg/mL SRM1650b OF or SRM2975 OF. The 8 h and 24 h air exposure equivalents were calculated based on the nanograms of PAHs per exposure and the amount of air a mouse would breathe in 8 h, which is 0.01m^3^, and 24 h, which is 0.03m^3^

**Table 2:** In vivo PM Exposure of PAHs

PAHs	ng PAH per in vivo PM exposure			ng PAH per in vivo PM exposure		
	SRM1650b	8 h	24 h	SRM2975	8 h	24 h
Phenanthrene	16.400	1640.0	546.67	4.3250	432.50	144.17
Anthracene	0.3900	39.000	13.000	0.00098000	0.97500	0.32500
Fluoranthene	12.025	1202.5	400.83	6.7250	672.50	224.17
Pyrene	11.025	1102.5	367.50	0.23500	23.500	7.8333
Benz[a]anthracene	1.6125	161.25	53.750	0.082500	8.2500	2.7500
Chrysene	3.3500	335.00	111.67	1.1450	114.50	38.167
Benzo[b]fluoranthene	1.6925	169.25	56.417	2.8750	287.50	95.833
Benzo[k]fluoranthene	0.57500	57.500	19.167	0.17475	17.475	5.8250
Benzo[e]pyrene	1.5900	159.00	53.000	0.28500	28.500	9.5000
Benzo[a]pyrene	0.31250	31.250	10.417	0.013250	1.3250	0.44167
Perylene	0.041750	4.1750	1.3917	0.014750	1.4750	0.49167
Indeno[1,2,3-cd]pyrene	1.1200	112.00	37.333	0.34250	34.250	11.4167
Benzo[g,h,i]perylene	1.5100	151.00	50.333	0.12225	12.225	4.0750
Dibenz[a,h]anthracene	0.096750	9.6750	3.2250	0.072000	7.2000	2.4000
Picene	0.12650	12.650	4.2167	0.10650	10.650	3.5500
Sum of PAHs	51.868 ng	5186.8 ng/m^3^	1728.9 ng/m^3^	16.528 ng	1652.8 ng/m^3^	550.94 ng/m^3^

The nanograms of each PAH in the 20 μL PM exposure were calculated based on a dose of 12.5 mg/mL SRM1650b PM or SRM2975 PM. The 8 h and 24 h air exposure equivalents were calculated based on the nanograms of PAHs per exposure and the amount of air a mouse would breathe in 8 h, which is 0.01m^3^ and 24 h, which is 0.03m^3^

#### Isolation of naïve T cells and T cell differentiation

Naïve CD4^+^ T cells were isolated by negative selection and purified from male or female adult C57BL/6 J WT, *Ahr*^*−/−*^, or *CypTKO* mice using CD4^+^ Isolation Kit (Miltenyi) in conjunction with QuadroMACS separator (Miltenyi). Media used for cultures was RPMI 1640 (Cell Gro) supplemented with Hepes buffer (Cell Gro), non-essential amino acids (Cell Gro), sodium pyruvate (Cell Gro), penicillin/streptomycin/glutamine (Cell Gro), 2-Mercaptoethanol (Life Technologies) and 5% FBS (Hyclone).

Purified naïve CD4^+^ T cells were plated in 96-well plates at 150,000 cells per well in 100 μL and stimulated with plate-coated anti-CD3 (1 μg/ml; R&D Systems) at 4 °C for 24 h and by soluble anti-CD28 (1 μg/mL, BD) added at time 0. Cells were differentiated under Th17 conditions (human TGF-β (5 ng/mL; R&D Systems), murine IL-6 (50 ng/mL; R&D Systems)), Th1 conditions (murine IL-12 (10 ng/mL; R&D systems), or Treg conditions (human TFG-β 5 ng/mL; R&D Systems) for 72 h (3 days) at 37 °C and 5% CO_2_. Different stimulating cytokines are required to generate different subsets of T cells from naïve T cells. All cultures included two positive controls, 6-formylindolo[3,2-b] carbazole (FICZ) (200 nM; Enzo Life Sciences), which is a tryptophan photoproduct and endogenous high affinity AHR ligand and β-naphthoflavone (BNF) another AHR ligand (2 μM; Sigma Aldrich). The positive controls were used to determine whether the differentiation cultures were prepared appropriately, and naïve cells responded and differentiated. All treatments were done in duplicate or triplicate on each 96-well plate.

#### PM treatments

Cells were treated with 5 doses of SRM1650b PM (NIST), SRM2975 PM (NIST) or phosphate buffered saline (PBS) control added to the culture at time 0. The treatments were in 100 μL, making the final volume in each well of the 96-well plate 200 μL. The doses were based on mass of PM per volume. The highest dose was 40 μg/mL PM and the lowest dose was 0.78 μg/mL PM. The doses were chosen to be 1:1000 less than the in vivo dose and in an effort to obtain a complete dose response higher doses and lower doses were added to complete the dose response. The conversion of doses in micrograms PM per milliliter of treatment to micrograms OC per milliliter of treatment so that the PM treatment can be compared to the OF (Additional file 1: Table S3). In addition, the mass of PM per cell in each well was calculated as well as the corresponding mass of OC per cell (Additional file 1: Table S3).

#### OF treatments

Cells were treated with 8 doses of SRM1650b OF and SRM2975 OF (Dr. James Schauer, WSLH, Madison, WI) and solvent controls (Dr. James Schauer, WSLH, Madison, WI) added to the culture at time 0. The treatments were in 100 μL, making the final volume in each well of the 96-well plate 200 μL. There was 0.5% of solvent, primarily DMSO, per well in 200 μL. The treatment doses chosen were based on organic carbon (OC) content because it is extractable and PM, OF, and PAH mixture treatments can be normalized to it. The highest dose was 10 μg/mL OC and the lowest dose was 0.00001 μg/mL OC. The conversion of doses in micrograms OC per milliliter of treatment to micrograms PM per milliliter of treatment so that the OC treatment can be compared to the PM (Additional file 1: Table S4). In addition, the mass of OC per cell in each well was calculated as well as the corresponding mass of PM per cell (Additional file 1: Table S4).

#### PAH mixture treatments

Cells were treated with 8 doses of SRM1650b PAH mixtures and SRM2975 PAH mixtures (Dr. James Schauer, WSLH, Madison, WI) and solvent controls (Dr. James Schauer, WSLH, Madison, WI) at time 0. The treatments were in 100 μL, making the final volume in each well of the 96-well plate 200 μL. The treatment doses chosen were based on organic carbon (OC) content because it is extractable and PM, OF, and PAH mixture treatments can be normalized to it. The highest dose was 10 μg/mL OC and the lowest dose was 0.00001 μg/mL OC. The concentration of each individual PAH was calculated for the highest dose of 10 μg/mL OC as well as the ng/PAH per cell at this dose (Additional file 1: Table S2).

#### In vitro dosing relevance

The cells are plated in a 96-well plate with 150,000 cells per well. The dose of PM per cell was calculated for the PM doses and ranges from 0.0001 to 0.17 picograms per cell (Additional file 1: Table S3). Additionally, the dose of PM per cell was calculated for the OF doses and ranges from 0.00002–229.70 pg PM/cell (Additional file 1: Table S4). In addition, the dose of OC per cell was calculated and ranges from 5.0E-09 to 0.05 pg OC/cell (Additional file 1: Table S4). The tables also provide the conversion from mass of PM to mass of OC. The doses chosen for this study are relatively low compared to doses used in other in vitro studies, however endpoints and cell types are different [57, 58].

#### Intracellular cytokine staining

Intracellular cytokine staining was conducted on day 3, after the T cells were cultured for 72 h. The cultured cells were stimulated with Cell Stimulation Cocktail (eBioscience) for 5 h. Brefeldin A 1000X (eBioscience) was added for the final 4.5 h. Cells were then fixed and permeabilized with Intracellular Fixation & Permeabilization Buffer (eBioscience) or Foxp3/Transcription Factor Staining Buffer Set (eBioscience) and intracellular cytokines overnight at 4 °C. Cells were stained with LIVE/DEAD Fixable Blue Dead Cell Stain Kit for UV Excitation (Invitrogen) or Ghost 780 (Tonbo) prior to fixation. Cells were stained with CD4 (BUV395; BD or PE, PeCy5; eBioscience) and TCRB (PeCy7; eBioscience) for extracellular markers. Cells were stained with IL-17A (FITC; eBioscience), IFNγ (eFluor450; eBioscience), and/or FOXP3 (eFluor450, eFluor660; eBioscience). Stained cells were analyzed on Fortessa (BD) or Attune NxT (Invitrogen). Data was analyzed using FlowJo software (TreeStar). Flow plots show cytokine producing cells as percent cytokine producing cells of CD4^+^TCRβ^+^ Live cells.

### Statistics

Statistics were analyzed using GraphPad Prism version 7. For both PM and OF in vitro analyses, the two variables tested were treatment and dose. For all in vitro experiments the question asked was: Is there an interaction between treatment and dose for the different samples? A D’Agostino-Pearson omnibus normality test was conducted to determine whether the statistics were parametric or nonparametric. A two-way repeated measure analysis of variance (ANOVA) followed by Dunnett’s multiple comparison test were performed with *p* value < 0.05. The treatments were SRM1650b, SRM2975, or control and the doses were the 5–8 doses tested.

For the in vivo EAE experiments, the question asked was is there an effect of treatment of clinical response? A D’Agostino-Pearson omnibus normality test was conducted to determine whether the statistics were parametric or nonparametric. For all OF experiments, an ordinary one-way ANOVA followed by a Holm-Sidak test of multiple comparisons was conducted with a *p* value ≤ 0.05. For all PM experiments, unpaired Mann Whitney T-test were conducted with a *p* value ≤ 0.05. For EAE ratio of daily weight over day 0 weight, a two-way ANOVA followed by Dunnett’s multiple comparison test was conducted with a *p* value ≤ 0.05. The treatments were SRM1650b, SRM2975, or control.

## Results

### Diesel exhaust PM samples worsen EAE in vivo

**Fig. 2:**
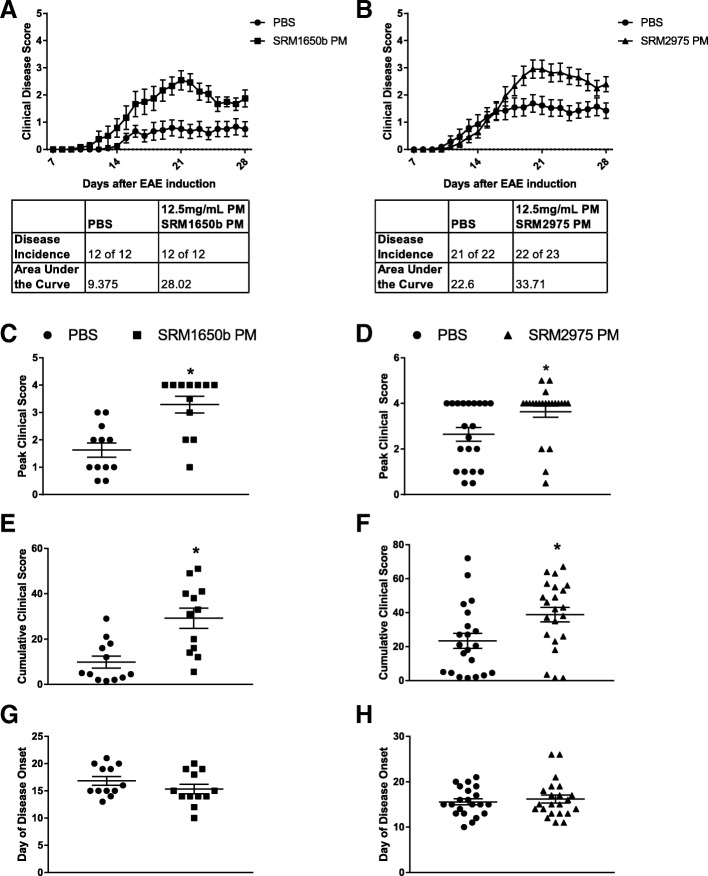
Diesel exhaust particles worsen severity of EAE in vivo. Age matched female WT (C57BL/6 J) were exposed intranasal to 12.5 mg/mL PM of SRM1650b PM, SRM2975 PM, or solvent control all diluted in PBS. The mice were dosed intranasal 8 times starting at day − 12 every 3 days until day 9 after induction. Mice were scored daily for severity of disease. **a**, **b** SRM1650b and SRM2975 PM worsen severity of EAE. **c**, **d** SRM1650b and SRM2975 PM significantly increased peak score. **e, f** Both DEPs significantly increased cumulative clinical score over the 28-day period. **g, h** Neither DEP influenced day of disease onset which represents the first day of a clinical score greater than 0. Results are mean ± SEM of (PBS *n* = 12) (SRM1650b PM *n* = 13) and (SRM2975 PM n = 13). Significant differences among groups (*p* < 0.05) are indicated by an asterisk. Abbreviations: SRM, standard reference materials; PM, particulate matter; PBS, phosphate buffered saline; EAE, experimental autoimmune encephalomyelitis; DEP, diesel exhaust particles

Previously, our lab published that an ambient urban dust PM, SRM1649b, could enhance Th17 differentiation in vitro in an AHR-dependent manner and in vivo [44]. Given this and that Th17 cells are central to the mechanism of many autoimmune disorders [45, 59–65], two additional SRMs were obtained from diesel exposures: SRM1650b (4-cylinder truck engine diesel) and SRM2975 (2-cylinder forklift engine diesel). SRM1650b and SRM2975 DEPs contain PAHs, some of which are strong AHR ligands. We hypothesized that intranasal exposure to diesel exhaust PM would worsen EAE in mice due to an enhanced effector T cell response. To test this hypothesis, mice received intranasal doses of 12.5 mg/mL PM of SRM1650b PM, SRM2975 PM, or PBS control starting at day − 12 of EAE induction and continuing every 3 days until day 9 after induction. On day 0, mice were IP injected with pertussis toxin and subcutaneously with MOG_35–55_ emulsion. A follow-up IP injection of pertussis toxin as a booster was given 44–52 h later. Mice were weighed, and clinical scores were recorded daily for 28 days (Fig. 2a and b). There was no significant reduction in weight loss observed over the course of 28 days for either SRM1650b PM or SRM2975 PM (Additional file 1: Fig. S2A and 2B). Both SRM1650b PM (Fig. 2a) and SRM2975 PM (Fig. 2b) significantly aggravated disease compared to vehicle control. SRM1650b PM and SRM2975 PM-treated mice had significantly higher peak clinical scores than control, (Fig. 2c and d) and both treatment groups had significantly higher cumulative clinical scores than control (Fig. 2e and f). There was no difference in day of disease onset for SRM1650b PM or SRM2975 PM (Fig. 2g and h).

#### SRM2975 OF worsens severity of EAE and SRM1650b OF has no effect on EAE

**Fig. 3:**
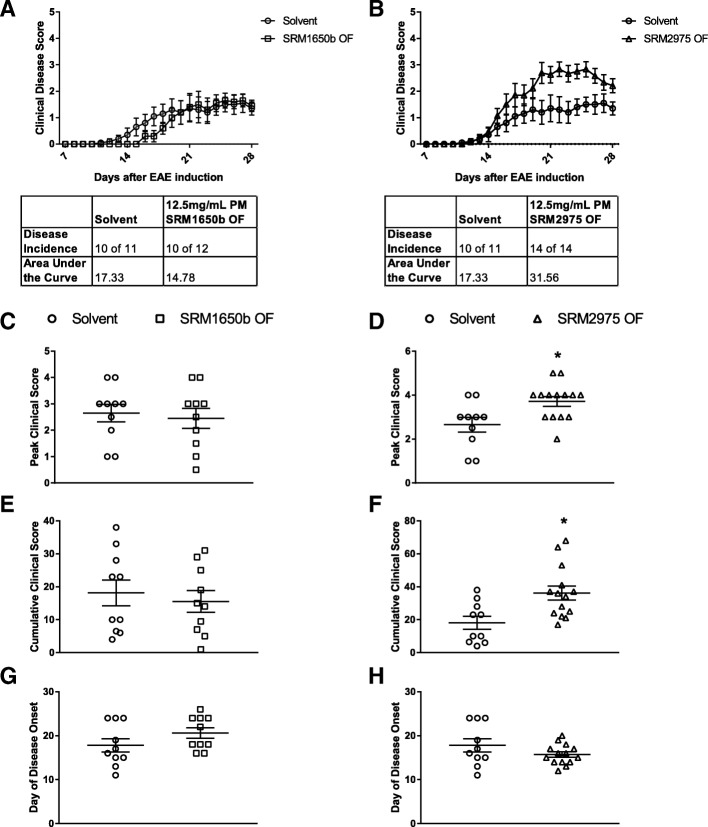
Organic fraction of diesel exhaust particles have differential effects on severity of EAE in vivo. Age matched female C57BL/6 J were exposed intranasally to 12.5 mg/mL PM of SRM1650b OF, SRM2975 OF, or PBS control. The mice were dosed intranasal 8 times starting at day − 12 and continuing every 3 days until day 9. Mice were scored daily for severity of disease. **a** SRM1650b OF had no effect on severity of EAE. **b** SRM2975 OF worsens severity of EAE. **c** SRM1650b OF does not increase peak clinical score. **d** SRM2975 OF significantly increased peak clinical score. **e** SRM1650b OF had no effect on cumulative clinical score. (F) SRM2975 OF significantly increased cumulative clinical score over the 28-day period. **g**, **h** Neither DEP OF influenced day of disease onset which represents the first day of a clinical score greater than 0. Results are mean ± SEM of (Solvent *n* = 10), (SRM1650b OF n = 10), and (SRM2975 OF *n* = 14). Significant differences among groups (*p* < 0.05) are indicated by an asterisk. Abbreviations: SRM, standard reference materials; PM, particulate matter; OF, organic fraction; PBS, phosphate buffered saline; EAE, experimental autoimmune encephalomyelitis; DEP, diesel exhaust particles

SRM1650b OF and SRM2975 OF were tested for their ability to worsen EAE. The mice were exposed to 12.5 mg/mL SRM1650b OF and SRM2975 OF intranasally every 3 days starting at day − 12 until day 9 post-induction. OF extracts and solvent were diluted into PBS at a mass of 250 μg PM or 12.5 mg/mL per dose. There was no significant reduction in weight loss observed over the course of 28 days for either SRM1650b OF or SRM2975 OF (Additional file 1: Figure S2C and 2D). SRM1650b OF had no effect on EAE at the concentrations measured (Fig. 3a, c, e, and g). In contrast, SRM2975 OF did aggravate disease (Fig. 3b). Mice that inhaled SRM2975 OF had significantly higher peak clinical scores and cumulative clinical scores as compared to solvent PBS control (Fig. 3d and f). SRM2975 OF had no effect on day of disease onset (Fig. 3h). Note that the samples and controls shown in this experiment are not comparable to that of the PM shown in Fig. 2 as these samples contain solvent that underwent a DCM/DMSO extraction and then was diluted into PBS. The solvent control referred to here is solvent diluted into PBS, which may have reduced total disease in the control and experimental mice.

### Diesel exhaust PM samples have differential AHR-mediated effects on Th17 differentiation in vitro

**Fig. 4:**
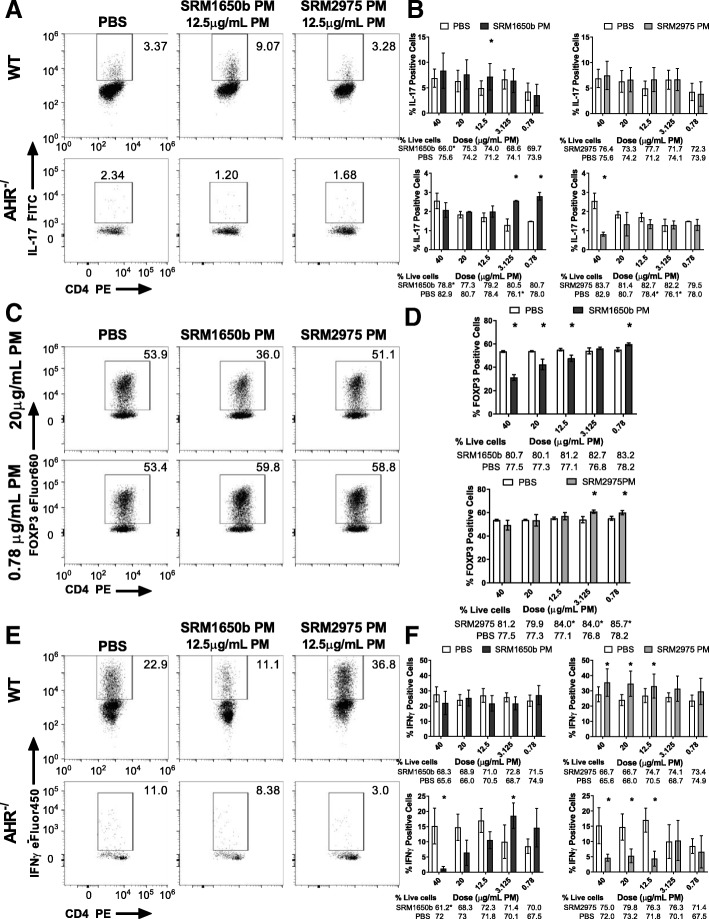
Diesel exhaust particles alter T cell differentiation in vitro. Naïve CD4^+^ T cells were isolated from WT (C57BL/6 J) or *Ahr*^−/−^ mice. At time 0, cells were exposed to a dose-response (5 doses) of SRM1650b PM, SRM2975 PM, or PBS control and cultured for 3 days. **a** Representative flow plots of WT (top) and *Ahr*^−/−^ (bottom) Th17 differentiation at 12.5 μg/mL PM, measured by percent IL-17 positive cells. **b** SRM1650b PM enhanced Th17 differentiation at 12.5 μg/mL PM compared to PBS control and SRM2975 PM had no effect on Th17 differentiation (top). In *Ahr*^−/−^ cells, SRM1650b PM significantly increased % IL-17 positive cells at the two lowest doses. SRM2975 PM suppressed Th17 differentiation in *Ahr*^−/−^ cells at 40 μg/mL PM (bottom). **c** Representative flow plots of WT Treg differentiation at 20 μg/mL PM (top) and 0.78 μg/mL PM (bottom), measured by percent FOXP3 positive cells. **d** SRM1650b PM suppresses Treg differentiation at 40, 20, and 12.5 μg/mL PM and enhances Treg differentiation at 0.78 μg/mL PM (top). SRM2975 PM enhances Treg differentiation at 3.125 and 0.78 μg/mL PM (bottom). **e** Representative flow plots of WT (top) and *Ahr*^−/−^ (bottom) Th1 differentiation at 12.5 μg/mL PM, measured by percent IFNγ positive cells. **f** SRM1650b PM had no effect on Th1 differentiation in WT cells (top). SRM2975 PM enhanced Th1 differentiation at 40, 20, and 12.5 μg/mL PM (top). In *Ahr*^−/−^ cells, SRM1650b PM suppressed Th1 differentiation at 40 and 20 μg/mL PM but enhanced Th1 differentiation at 3.125 μg/ml PM (bottom). SRM2975 PM suppresses Th1 differentiation in *Ahr*^−/−^ cells at 40, 20, 12.5 μg/mL PM (bottom). Results are mean ± SEM of (WT Th17 *n* = 5), (*Ahr*^−/−^ Th17 *n* = 2), (WT Treg n = 2), (WT Th1 n = 5), and (*Ahr*^−/−^ Th1 n = 2). Significant differences among groups (*p* < 0.05) are indicated by an asterisk. Abbreviations: AHR, aryl hydrocarbon receptor; SRM, standard reference materials; PM, particulate matter; PBS, phosphate buffered saline

Given that EAE is driven by Th17 and Th1 cells [45, 48, 66], we hypothesized that SRM1650b PM and SRM2975 PM would enhance Th17 differentiation in an AHR-mediated dose-dependent manner. Naïve CD4 positive T cells were isolated from spleens of C57BL/6 J WT mice and exposed to a dose-response of SRM1650b PM, SRM2975 PM, or PBS on day 0, cultured under Th17 conditions for 3 days, and flow cytometry was performed to measure the percent of IL-17 expressing cells. SRM1650b PM enhanced Th17 differentiation at 12.5 μg/ml PM, measured by percent interleukin (IL)-17 positive cells, (Fig. 4a (top middle) and 4B (top left)) whereas SRM2975 PM had no effect on Th17 differentiation over PBS control (Fig. 4a (top right) and 4B (top right)).

Next, we tested whether SRM1650b PM-enhanced Th17 differentiation was AHR-dependent. Naïve CD4 positive T cells were isolated from *Ahr*^−/−^ mice. SRM1650b PM at the 12.5 μg/mL dose no longer enhanced Th17 differentiation, suggesting this effect was AHR-dependent (Fig. 4a (bottom middle) and 4B (bottom left)). Interestingly, treatment with SRM1650b PM in Th17 *Ahr*^−/−^ cells resulted in an enhanced Th17 differentiation at the two lowest doses (Fig. 4b (bottom left)). Additionally, SRM2975 PM reduces Th17 differentiation in *Ahr*^−/−^ cells at the highest dose tested: 40 μg/ml PM (Fig. 4b (bottom right)). The PBS treated *Ahr*^*−/−*^ cells exhibited a significant reduction in percent live cells at 3.125 μg/mL PM for SRM1650b PM (Fig. 4 (bottom left)) and 12.5 and 3.125 μg/mL PM for SRM2975 PM (Fig. 4 (bottom right)). This finding suggests AHR is important for generation of Th17 cells at high doses of SRM2975 PM exposure.

### Diesel exhaust PM samples have differential effects on Th1 and Treg differentiation in vitro

The AHR plays a critical role in shifting the balance between effector T cell responses and regulatory T cell responses [40, 41, 67, 68]. *Ahr* is most highly expressed in Th17 cells, is moderately expressed in T regulatory type 1 (Tr1) cells and forkhead box P3 (FOXP3) ^+^ Treg cells and has low expression in Th1 cells [41, 69]. Given the role of AHR in modulating T cell responses and its expression in other T cell subsets, we tested the ability of DEPs to enhance Th1 and FOXP3^+^ Treg differentiation. SRM1650b PM suppressed Treg differentiation at multiple high doses, measured by percent FOXP3 positive cells (Fig. 4c (top middle) and 4D (top)). SRM1650b PM enhanced Treg differentiation at the lowest dose tested (Fig. 4c (bottom middle) and 4D (top)) SRM2975 PM increased Treg differentiation at the two lowest doses tested: 3.125 and 0.78 μg/ml PM (Fig. 4d (bottom)). However, SRM2975 PM decreased the percent live cells at 12.5, 3.125, and 0.78 μg/mL PM (Fig. 4d (bottom)), suggesting the enhanced differentiation at low doses could be an artifact of differences in live cells.

In contrast to their effects on Treg differentiation, SRM1650b PM had no significant effect on Th1 differentiation measured by percent interferon gamma (IFNγ) producing cells (Fig. 4e (top middle) and 4F (top left)) and SRM2975 PM increased Th1 differentiation at the 3 highest doses tested (Fig. 4e (top left) and 4F (top right)). Using naïve CD4 positive *Ahr*^−/−^ T cells, SRM1650b PM reduced Th1 differentiation at 40 μg/ml PM and enhanced Th1 differentiation at 3.125 μg/ml PM (Fig. 4f (bottom left)). This indicates that AHR is important for generation of Th1 cells at high doses of SRM1650b PM exposure and suppressing Th1 differentiation at lower doses. SRM2975 PM suppressed IFNγ producing cells at 40, 20, and 12.5 μg/ml PM (Fig. 4e (bottom right) and 4F (bottom right)).

### Diesel exhaust OF samples enhance Th17 differentiation in vitro

**Fig. 5:**
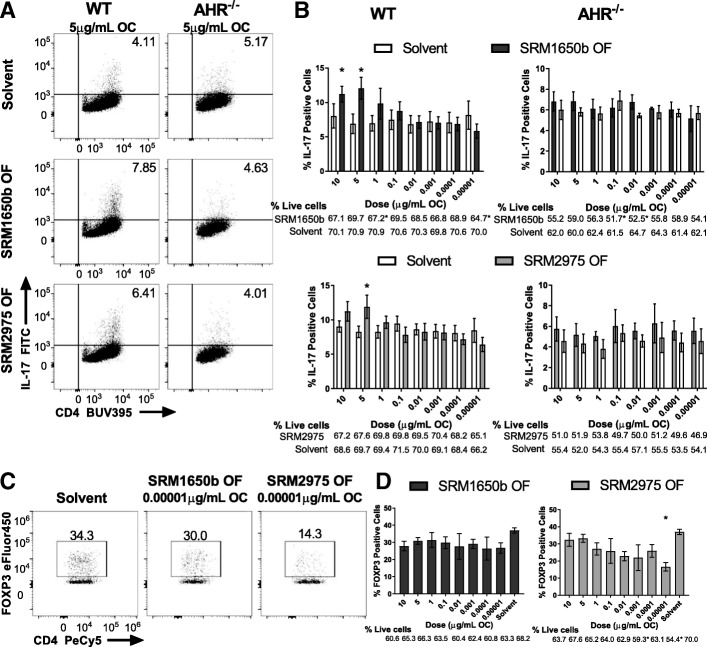
Organic fraction of diesel exhaust particles impact T cell differentiation in vitro. Naïve CD4^+^ T cells were isolated from WT (C57BL/6 J) or *Ahr*^−/−^ mice. At time 0 cells were exposed to a dose-response (8 doses) of SRM1650b OF, SRM2975 OF, or solvent control and cultured for 3 days. **a** Representative flow plots of WT (left) and *Ahr*^−/−^ (right) Th17 differentiation at 5.0 μg/mL OC, measured by percent IL-17 positive cells. **b** SRM1650b OF enhanced Th17 differentiation at 10 and 5 μg/mL OC compared to solvent control (top left) and SRM2975 OF enhanced Th17 differentiation at 5μg/mL OC (bottom left). In *Ahr*^−/−^ cells, SRM1650b OF lost theTh17 differentiation effect (top right) and SRM2975 OF had no effect on Th17 differentiation (bottom right). **c** Representative flow plots of WT Treg differentiation at 0.00001 μg/mL OC measured by percent FOXP3 positive cells. **d** SRM1650b OF has no effect on Treg differentiation (top). SRM2975 OF suppresses Treg differentiation at 0.00001 μg/mL OC (bottom). Results are mean ± SEM of (WT Th17 SRM1650b OF n = 5), (*Ahr*^−/−^ Th17 SRM1650b OF *n* = 4), (WT Th17 SRM2975 OF *n* = 6), (*Ahr*^−/−^ Th17 SRM2975 OF *n* = 3), and (WT Treg SRM1650b OF and SRM2975 OF n = 4), Significant differences among groups (*p* < 0.05) are indicated by an asterisk. Abbreviations: AHR, aryl hydrocarbon receptor; SRM, standard reference materials; OF, organic fraction; OC, organic carbon

Previously, our lab showed that the total OF of SRM1649b PM, an ambient urban dust particulate, enhanced Th17 differentiation to a similar degree as PM [44]. Given this, we tested the OF of DEP samples in an in vitro Th17 differentiation assay and in vivo in EAE. Naïve CD4 positive T cells under Th17 conditions were exposed to a dose-response of SRM1650b OF, SRM2975 OF, or solvent control on day 0. Solvent control is primarily DMSO but has undergone a DCM/DMSO extraction and hexane additions altering the composition. Both DEPs enhanced Th17 differentiation compared to solvent control as measured by percent IL-17 positive cells, although SRM1650b OF enhanced Th17 differentiation at 10 and 5 μg/ml organic carbon (OC) (Fig. 5a (middle left) and 5B (top left)), whereas SRM2975 OF only enhanced Th17 differentiation at 5 μg/ml OC (Fig. 5a (bottom left) and 5B (bottom left)). Enhanced Th17 differentiation by both DEPs was AHR-dependent (Fig. 5a (middle and top right) and 5B (top right and bottom right)).

### Diesel exhaust OF sample SRM2975 suppresses Treg differentiation in vitro

AHR is important in regulating the balance between Th17 and FOXP3^+^ Treg cells. Recent evidence shows that potency of ligand and extent of activation of the AHR can result in different ligands leading to different T cell responses and effector function [67]. Due to the established role of AHR in Tregs, we investigated the effect of DEP OF on Treg differentiation in vitro. SRM1650b had no measurable effect on Treg differentiation at the doses tested (Fig. 5c (middle) and 5D (left)). SRM2975 OF suppressed Treg differentiation at the lowest dose tested (0.00001 μg/mL OC) compared to solvent control (Fig. 5c (bottom) and 5D (right)). Again, the reduced differentiation at low doses by SRM2975 OF could be an artifact of increased cell death observed at 0.00001 μg/mL OC (Fig. 5d (right)).

### SRM1650b PAH mixtures enhance Th17 differentiation in an AHR-dependent manner and SRM2975 PAH mixtures suppress Treg differentiation at low doses

**Fig. 6:**
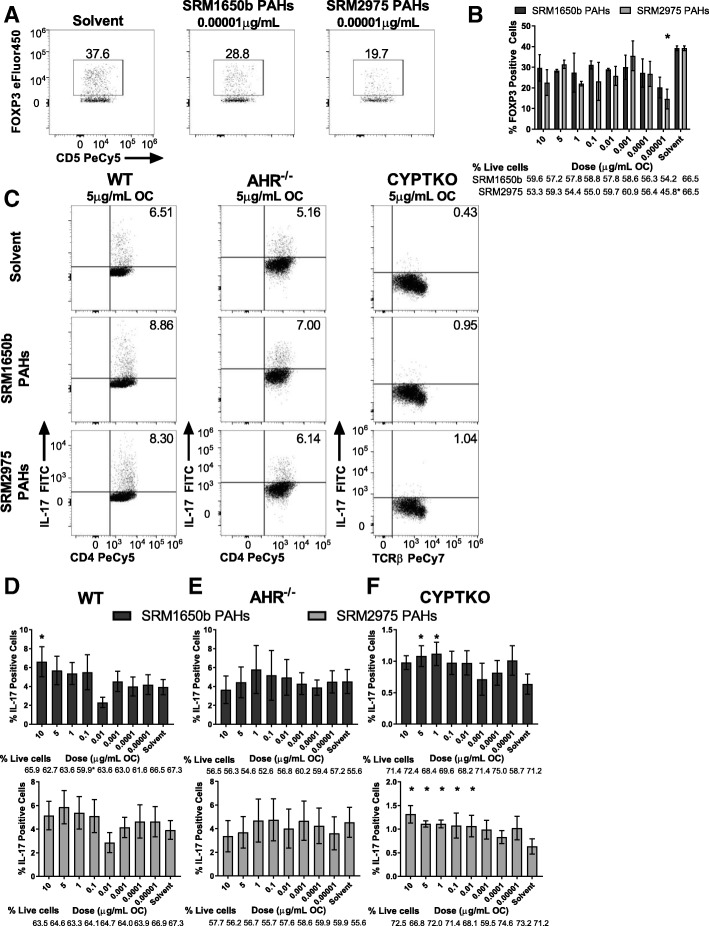
Diesel exhaust PAH mixtures alter Treg differentiation and have AHR and CYP dependent effects on Th17 differentiation. Naïve CD4^+^ T cells were isolated from WT (C57BL/6 J), *Ahr*^−/−^, or *CypTKO* mice. At time 0 cells were exposed to a dose-response (8 doses) of SRM1650b PAH mixtures, SRM2975 PAH mixtures, or solvent control and cultured for 3 days. **a** Representative flow plots of WT Treg differentiation at 0.00001 μg/mL OC, measured by percent FOXP3 positive cells. **b** SRM2975 PAH mixtures suppressed Treg differentiation at the lowest dose compared to solvent control. **c** Representative flow plots of WT (left), *Ahr*^−/−^ (middle), and *CypTKO* (right) at 5 μg/ml OC. **d** SRM1650b PAH mixtures enhanced Th17 differentiation at 40 μg/ml OC (top) and SRM2975 PAH mixtures had no effect (bottom). **e** In *Ahr*^−/−^ cells, SRM1650b (top) and SRM2975 (bottom) PAH mixtures had no effect on percent IL-17 positive cells. **f** In *CypTKO* cells, SRM1650b PAH mixtures enhanced Th17 differentiation at 5μg/ml OC (top) and SRM2975 PAH mixtures enhanced Th17 differentiation at the three highest doses (bottom). Results are mean ± SEM of n = 2 (WT Treg), *n* = 7 (WT Th17), n = 4 (*Ahr*^−/−^ Th17), and n = 6 (*CypTKO* Th17). Significant differences among groups (*p* < 0.05) are indicated by an asterisk. Abbreviations: AHR, aryl hydrocarbon receptor; CYP, cytochrome P450; TKO, triple knockout; SRM, standard reference materials; PAH, polycyclic aromatic hydrocarbons; OC, organic carbon

The AHR serves as an environmental sensor responding to exogenous ligands, including PM components such as PAHs. [38, 70]. The DEPs contain high levels of PAHs compared to other components. To test the hypothesis that the specific PAHs present in each DEP were driving the effects on T cell differentiation in vitro PAH mixtures that replicate the PAH milieu of the OF extracts of SRM1650b and SRM2975 were synthesized and assayed in the naïve CD4 T cell assay under Th17 differentiation conditions (Additional file 1: Table S2). SRM2975 PAH mixtures suppressed Treg differentiation, measured by percent FOXP3 positive cells, at the lowest dose tested, however there was a significant difference between percent live cells of SRM2975 PAH treated and solvent control at the lowest dose tested (Fig. 6a and b). SRM1650b PAH mixtures enhanced Th17 differentiation at the highest dose tested 10 μg/mL OC (Fig. 6c (middle left) and 6D (top)). SRM2975 PAH mixtures had no measurable effect on Th17 differentiation at the doses tested (Fig. 6c (bottom left) and 6D (bottom)).

In *Ahr*^−/−^ naïve T cells, SRM1650b had no observable effect on Th17 differentiation (Fig. 6c (middle middle) and 6E (top)). Additionally, SRM2975 PAH mixtures did not have an AHR-dependent effect on Th17 differentiation (Fig. 6c (bottom middle) and 6E (bottom)). This suggests that SRM1650b elicits in vivo effects through AHR, whereas SRM2975 may not.

### Metabolism by cytochrome P450 (CYP) enzymes alters T cell responses by diesel exhaust PAH mixtures

Cytochrome P450 (CYP) 1A1, CYP1A2, and CYP1B1 enzymes are all canonical downstream targets of AHR activation and are essential for phase 1 metabolism of xenobiotic toxicants [71, 72]. PAHs are high affinity AHR ligands and are readily metabolized by these CYP enzymes. Given this, and the fact that SRM2975 PAH mixtures do not enhance Th17 differentiation, the role CYP enzymes play in response of PAH mixtures was tested. We hypothesized that SRM2975 PAH mixtures would enhance Th17 differentiation in the absence of the metabolizing enzymes and loss of CYP enzymes would not impact SRM1650b PAH mixture responses. Naïve CD4 positive T cells under Th17 conditions were isolated from spleens of *Cyp1a1*^−/-^*Cyp1a2*^−/-^*Cyp1b1*^−/−^ (*CypTKO*) mice and exposed to a dose-response of SRM1650b, SRM2975 PAH mixtures or solvent control. In *CypTKO* T cells SRM1650b PAH mixtures enhanced Th17 differentiation at 5, and 1 μg/ml OC (Fig. 6c (middle right) and 6F (top)) and SRM2975 PAH mixtures enhanced Th17 differentiation at the 5 highest doses tested (Fig. 6c (bottom right) and 6F (bottom)). SRM1650b PAH mixtures appear to act through the AHR to enhance Th17 differentiation at high doses and at low doses CYP enzymes break down PAHs and eliminate their effect. In contrast, CYPs likely break down SRM2975 PAHs quickly and do not allow for enhanced Th17 differentiation.

## Discussion

Autoimmune diseases are characterized by a loss of immunological tolerance to self-antigens and reflect an imbalance of effector and regulatory immune responses. The pathology of disease can be mediated by self-reactive, effector T cells that contribute to the loss of tolerance by suppressing regulatory responses [45]. Under pathologic conditions, effector Th17 cells can induce inflammatory cytokines and chemokines that recruit effector Th1 cells to the target tissues [45]. Normally, Treg cells modulate this inflammatory response, but in disease states the overwhelming proinflammatory milieu generated by the effector T cells can render them ineffective [45, 48, 66]. Another possibility is that the Treg cells are dysfunctional or absent and cannot modulate the inflammatory response [6, 46, 73, 74]. We postulate that exposure to PM throws off the balance of effector and regulatory cells and puts some patients at risk for autoimmune disease.

Past regulatory efforts to reduce PM emissions and atmospheric concentrations have not eliminated the impact of human exposures to particulate matter and highlight the need to further reduce sources of atmospheric PM [1, 2]. The need for additional control measures raises the question as to where further efforts should continue along the same air quality management strategies to reduce the total mass of PM or to pursue more targeted strategies to reduce specific sources, such as diesel exhaust, or components of PM. Atmospheric PM is currently regulated by total mass based on the assumption that all PM is equally toxic [75, 76]. Individual particle characteristics or components are not considered [75, 76]. The findings of this study would suggest this is not an efficacious way to regulate PM to reduce adverse health outcomes. This study focused on identifying the roles of total PM mass and the active components of PM in shifting effector T cell responses and which molecular pathways are responsible for disease responses, with a goal of understanding mechanisms behind PM-induced autoimmunity and developing strategies to reduce environmental-induced pathology.

The AHR pathway was a focus of this study given its modulatory role in the balance between effector and regulatory T cells [40, 41, 67, 68]. Some AHR ligands ameliorate autoimmunity by enhancing Treg differentiation, while other AHR ligands have been shown to aggravate autoimmunity by augmenting Th17 differentiation [40, 41]. This phenomenon has raised an interesting question: What characteristics of AHR-ligand interactions dictate the biologic response in T cell differentiation? Recent data would suggest that the extent and duration of AHR activation, not innate features of the ligands themselves is the primary driving factor [67]. This study further explores the AHR-ligand relationship in the context of PM pollution to determine how and which AHR ligands present in PM aggravate disease.

**Fig. 7:**
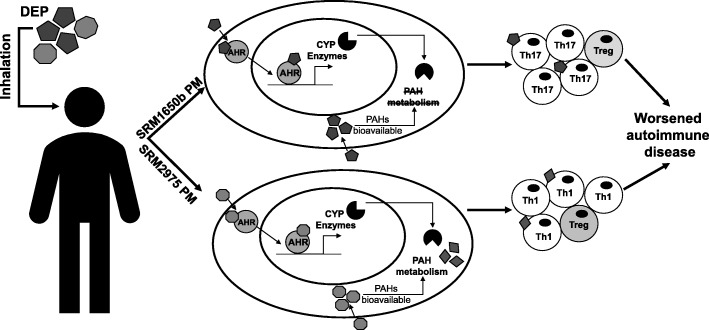
Summary of DEP mediated autoimmune disease. Two DEPs, SRM1650b from a 4-cylinder diesel engine, and SRM2975 from a 2-cylinder diesel engine, were tested for the effects on T cell differentiation and autoimmune disease. SRM1650b enters the T cell, binds AHR, which then translocate to the nucleus and binds DNA, driving transcription of CYP enzymes (top). SRM1650b enhances Th17 differentiation in an AHR-dependent manner and worsens autoimmune disease (top). Based on the in vivo EAE data, SRM1650b requires the particle to aggravate autoimmune disease because of bioavailability of the PAHs and their ability to activate the AHR. Similarly, SRM1975 enters the T cell, binds AHR, moves to the nucleus, binds DNA, and drives transcription of CYP enzymes (bottom). SRM2975 enhances Th1 differentiation in an AHR-dependent manner and worsens autoimmune disease (bottom). Based on the in vivo EAE data demonstrating SRM2975 worsens autoimmune disease in PM and OF forms and the in vitro data showing a role of CYP enzymes in T cell differentiation, metabolism of SRM2975 plays a role in its ability to worsen autoimmune disease in that CYP metabolism of PAHs may lead to more potent intermediate that drives the response in vivo. Additionally, in the presence of PAHs and AHR activation, enhanced effector differentiation by both samples results in increase in Th17 or Th1 cells and a reduction in Treg cells. However, when PAHs are diluted at low doses, enhanced effector differentiation is no longer observed, and the balance is switched to enhanced Treg differentiation. Abbreviations: SRM, standard reference materials; DEPs, diesel exhaust particles; AHR, aryl hydrocarbon receptor; CYP, cytochrome P450; PAH, polycyclic aromatic hydrocarbons

We tested the hypothesis that the PAHs present in two DEP source samples and their derivative OFs would enhance effector T cell differentiation and aggravate autoimmunity after inhalation via the AHR pathway. First, we tested the ability of diesel PMs to aggravate disease in an experimental model of murine EAE. Inhalation of both diesel PMs significantly aggravated EAE in mice. Since both diesel PM aggravated EAE, we next tested whether the OF of the two diesel PM would aggravate EAE. Unlike the finding with PM, where both samples worsened disease, inhalation of SRM2975 OF worsened severity of disease, but SRM1650b OF had no effect on EAE. These differences are likely due to the concentration, bioavailability, and duration of activity of the active components present in the OF. More specifically, SRM1650b may require the particle to prevent metabolism of the PAHs in the mixture whereas SRM2975 does not because of the higher concentration of PAHs and their specific characteristics, allowing their metabolism with the intermediates readily driving the response (Fig. 7). The particulate matter may also disrupt the metabolism of active components, thus extending their presence and altering their biological effects. These findings make it clear that the particulate matrix itself has an important role in determining disease, not simply the total mass of PM which assumes same toxicity for all samples.

To understand why PM and OF exhibited differential EAE effects, the ability of the diesel PMs and derivative OFs to enhance T cell differentiation were tested in vitro. Both DEPs suppressed Treg differentiation at high doses, but this suppression was reversed at low doses and DEPs began to enhance Treg differentiation (Table 3). At high doses, the PAH concentrations achieved are sufficient to enhance effector differentiation. When the samples are diluted the PAHs are at lower concentrations, activating the AHR to a lesser degree, and effector differentiation is no longer enhanced allowing Treg differentiation to be enhanced.

**Table 3:** Summary of in vitro T cell differentiation results

In vitro	Genotype	WT		Ahr^−/−^		CypTKO	
	Dose	High	Low	High	Low	High	Low
Th17	SRM1650b PM	↑	–	–	↑		
	SRM2975 PM	–	–	↓	–		
Treg	SRM1650b PM	↓	↑				
	SRM2975 PM	–	↑				
Th1	SRM1650b PM	–	–	↓	↑		
	SRM2975 PM	↑	–	↓	–		
Th17	SRM1650b OF	↑	–	–	–		
	SRM2975 OF	↑	–	–	–		
Treg	SRM1650b OF	–	–				
	SRM2975 OF	–	↓				
Treg	SRM1650b PAHs	–	–				
	SRM2975 PAHs	–	↓				
Th17	SRM1650b PAHs	↑	–	–	–	↑	–
	SRM2975 PAHs	–	–	–	–	↑	↑

Both DEP PM enhanced effector T cell differentiation in an AHR-dependent manner and suppressed regulatory T cell differentiation at high doses. Both DEP OF enhanced effector T cell differentiation in an AHR-dependent manner at high doses and SRM2975 OF suppressed regulatory differentiation at low doses. SRM1650b PAH mixtures enhanced Th17 differentiation at high doses in an AHR-dependent manner and SRM2975 PAH mixtures did not. In the absence of CYP metabolizing enzymes, both diesel PAH mixtures enhanced Th17 differentiation at high doses and SRM2975 maintained this effect at low doses. Symbols: ↑ increase, ↓ decrease, − no change

SRM1650b PM enhanced Th17 differentiation and SRM975 PM enhanced Th1 generation in an AHR-dependent manner, suggesting that although these DEPs drive T cell differentiation towards different T cell fates they both ultimately lead to inflammatory responses. Even though both samples tested are DEP samples, they elicit their T cell effects by enhancing different T cell effector pathways. This could also explain why the PM and OF behave differently. The importance of the ultimate balance of T cell subsets in immune response has been well established and it has been shown that IFNγ production by Th1 cells suppresses the generation of Th17 cells [45–50, 77]. We observed that SRM1650b PM enhances Th17 differentiation and suppresses Th1 differentiation at high doses in an AHR-dependent manner, but SRM2975 PM does the opposite (Table 3).

When AHR is lost, the enhanced effector differentiation is also lost. At low doses, AHR activation now enhances regulation and suppresses effector T cell differentiation. This emphasizes that PM has dose-dependent effects that are likely due to particulate matrices altering availability of active components and ultimately biological effects.

Our data suggest that DEPs are causing aggravation of disease by driving different inflammatory responses; SRM1650b driving Th17 and SRM2975 driving Th1 may worsen severity disease by effecting initiation (former) versus progression (latter) of disease. Our in vitro differentiation data show diesel PM-mediated enhancement of effector T cell differentiation is AHR-dependent by SRM1650b which highlights a role for AHR in driving aggravated EAE after PM exposure and dependent on metabolism by CYP enzymes for SRM2975 suggesting bioavailability may play an important role in determining immunological effects.

The in vitro PM data showed that AHR-mediated effects of DEPs on T cell differentiation are dose-dependent. This dose-dependence may be influenced by the bioavailability of active components on the particulate, so we next determined if the active components of the PM samples are also present in the OF. SRM2975 OF suppressed Treg differentiation at low doses and both diesel OFs enhanced Th17 differentiation in an AHR-dose-dependent manner (Table 3). We found that that active component(s) present in PM are also present in the OF. The observed dose-dependency of the PM and OF-mediated effects on T cell differentiation in vitro suggest that the concentration of active component(s) changes with dose resulting in differential, dose- dependent effects. The notion that PM and OF can elicit differential effects yet share the AHR pathway is important to recognize when trying to identify specific active components. The dose of individual components in the context of the whole mixture must be considered when identifying the responsible constituents.

Our PM and OF in vitro data suggest active component(s), not total mass, drive differential effector T cell responses towards inflammatory responses. If total mass was sufficient to define inflammatory responses by PM and OF in vitro, both would elicit the same inflammatory responses, which is not the case.

To identify the active component in the DEPs, synthetic PAH mixtures that replicate the milieu of PAHs measured in DEP samples were tested in vitro. SRM1650b PAH mixtures enhanced Th17 differentiation in an AHR-dependent manner and SRM2975 PAH mixtures had no effect on Th17 differentiation in WT or *Ahr*^−/−^ cells (Table 3). In *CypTKO* naïve T cells, SRM1650b PAH mixtures enhanced Th17 differentiation at two doses and SRM2975 PAH mixtures enhanced Th17 differentiation at the five highest doses tested (Table 3). These results exemplify the importance of the specific chemical make-up of mixtures and the importance of CYP-mediated metabolism of AHR ligands in these mixtures that influence T cell responses in vitro*.*

## Conclusions

Predicting biologic effects of complex mixtures is made difficult by the variable availability of the components to the many different cell types that harbor the AHR, a ubiquitous receptor. This has been a challenge faced by most investigators studying the AHR and its role in human physiology and disease. This is further influenced by the route of exposure, inflammatory milieu where the binding takes place, and metabolism of the ligands by CYP enzymes. The data presented here shed light on some important questions and support the hypothesis that ligands of the AHR found in PM can aggravate autoimmune disease after inhalation. This is extremely important, given the strong epidemiologic data linking PM to increases in disease incidence and lack of any clear mechanisms explaining this relationship. We have found that the active components of PM, not the total mass, are the crucial factor mediating the biological responses. The bioavailability of these components and the particulate matrix within which they reside have important ramifications for disease. In complicated in vivo autoimmune responses, like those in EAE, different mixtures of PM yield different responses and the presence of the particle is important for a subset of mixtures. A potential mechanism for this variability is the ability of particles to influence the availability and metabolism of active PM components thereby varying the duration of exposure.

It is becoming increasingly clear that not all PM and their fractions aggravate autoimmune disease through the same molecular pathway and that all PM fractions do not elicit the same effect. Our data support the concept that each exposure has specific potential for autoimmune aggravation and that the nature of the particulate is important for activity in some mixtures but not all. Although we can do analysis of in vitro responses, given the differences in the chemicals involved in these samples, the potential different mechanisms, and the genetic differences of the recipients, each sample should be tested in vivo to develop a catalog of different samples and potential pathologic responses. Given the disparate findings with different fractions of PM, we think interrogation of these differences will be necessary to uncover the specific active components of each exposure and their potential to cause autoimmune disease. Only then will it be reasonable to propose targeted remediation and avoidance protocols to stem the tide of autoimmunity that is growing in populations facing air pollution.

## Additional files

Additional file 1: **Figure S1.** Endotoxin test on PM samples. PM samples were tested for the presence of endotoxin. (A) A standard curve was generated based on manufacturer’s protocol. (B) The standard curve was then used to determine endotoxin concentration of each sample. Both SRM1650b and SRM2975 PM levels were below 0.1 EU/mL indicating very low levels of endotoxin. **Figure S2.** EAE Weight Ratios. The mice were weighed on day 0 prior to induction of disease. After disease induction, mice were weighed starting on day 7 daily until day 28. The ratio of daily weight over day 0 weight was calculated. (A) No significant weight loss was observed at any day during the course of disease for SRM1650b PM, (B) SRM2975 PM, (C) SRM1650b OF, or (D) SRM2975 OF. **Table S1.** PAH concentrations in OF extracts. PAH concentrations of 15 EPA PAHs of concern for SRM1650b and SRM2975 OF. The ng of each PAH that was extracted using the DCM/DMSO solvent extraction was measured. The PAH concentrations are shown in ng of individual PAHs present in the highest 10 μg/mL OC exposure of the OF. Additionally, ng of PAH per cell in the highest 10 μg/mL OC exposure was also calculated. **Table S2.** PAH mixture PAH concentrations. PAH concentrations of 15 EPA PAHs of concern for SRM1650b and SRM2975. The PAH concentrations were measured in ug/mL of PAH extracted as well as ng of individual PAHs present in the highest 10 μg/mL OC exposure. Additionally, ng of PAH per cell at the highest 10 μg/mL dose was calculated. **Table S3.** PM in vitro dose conversion. This table converts the PM doses based on mass of PM to mass of organic carbon. Additionally, mass of PM and mass of OC per cell are calculated. **Table S4.** OF in vitro dose conversion. This table converts the OF and PAH mixture doses based on organic carbon to a measure based on mass of PM. Additionally, mass of OC and mass of PM per cell are calculated. (PDF 767 kb)

## Abbreviations

AHR: Aryl hydrocarbon receptor
*Ahr*^*−/−*^: Aryl hydrocarbon receptor null gene
ANOVA: Analysis of variance
CFA: Complete Freud’s adjuvant
CNS: Central nervous system
CYP: Cytochrome P450
*CypTKO*: Cytochrome P450 1a1, 1a2, 1b1 null genes
DCM: Dichloromethane or methylene chloride
DEP(s): Diesel exhaust particle(s)
DMSO: Dimethyl sulfoxide
EAE: Experimental autoimmune encephalomyelitis
EPA: Environmental Protection Agency
FOXP3: Forkhead box P3
IFNγ: Interferon gamma
IL: Interleukin
IP: Intraperitoneal
MOG: Myelin oligodendrocyte glycoprotein
MS: Multiple sclerosis
NIST: National Institute of Standards and Technology
OC: Organic carbon
OF: Organic fraction
PAHs: Polycyclic aromatic hydrocarbons
PBS: Phosphate buffered saline
PM: Particulate matter
SMPH: School of Medicine and Public Health
SRM(s): Standard reference material(s)
Th: T helper
Tr1: T regulatory type 1
WSLH: Wisconsin State Lab of Hygiene
WT: Wild-type
