# Supplementary material for: Differential effects of diesel exhaust particles on T cell differentiation and autoimmune disease
Source: Part Fibre Toxicol. 2018 Aug 24;15:35. doi: 10.1186/s12989-018-0271-3 (PMC6109291; doi:10.1186/s12989-018-0271-3)
Supplement: Supplementary file 1 — Figure S1. Endotoxin test on PM samples. PM samples were tested for the presence of endotoxin. (A) A standard curve was generated based on manufacturer’s protocol. (B) The standard curve was then used to determine endotoxin concentration of each sample. Both SRM1650b and SRM2975 PM levels were below 0.1 EU/mL indicating very low levels of endotoxin. Figure S2. EAE Weight Ratios. The mice were weighed on day 0 prior to induction of disease. After disease induction, mice were weighed starting on day 7 daily until day 28. The ratio of daily weight over day 0 weight was calculated. (A) No significant weight loss was observed at any day during the course of disease for SRM1650b PM, (B) SRM2975 PM, (C) SRM1650b OF, or (D) SRM2975 OF. Table S1. PAH concentrations in OF extracts. PAH concentrations of 15 EPA PAHs of concern for SRM1650b and SRM2975 OF. The ng of each PAH that was extracted using the DCM/DMSO solvent extraction was measured. The PAH concentrations are shown in ng of individual PAHs present in the highest 10 μg/mL OC exposure of the OF. Additionally, ng of PAH per cell in the highest 10 μg/mL OC exposure was also calculated. Table S2. PAH mixture PAH concentrations. PAH concentrations of 15 EPA PAHs of concern for SRM1650b and SRM2975. The PAH concentrations were measured in ug/mL of PAH extracted as well as ng of individual PAHs present in the highest 10 μg/mL OC exposure. Additionally, ng of PAH per cell at the highest 10 μg/mL dose was calculated. Table S3. PM in vitro dose conversion. This table converts the PM doses based on mass of PM to mass of organic carbon. Additionally, mass of PM and mass of OC per cell are calculated. Table S4. OF in vitro dose conversion. This table converts the OF and PAH mixture doses based on organic carbon to a measure based on mass of PM. Additionally, mass of OC and mass of PM per cell are calculated. (PDF 767 kb) [file 12989_2018_271_MOESM1_ESM.pdf]

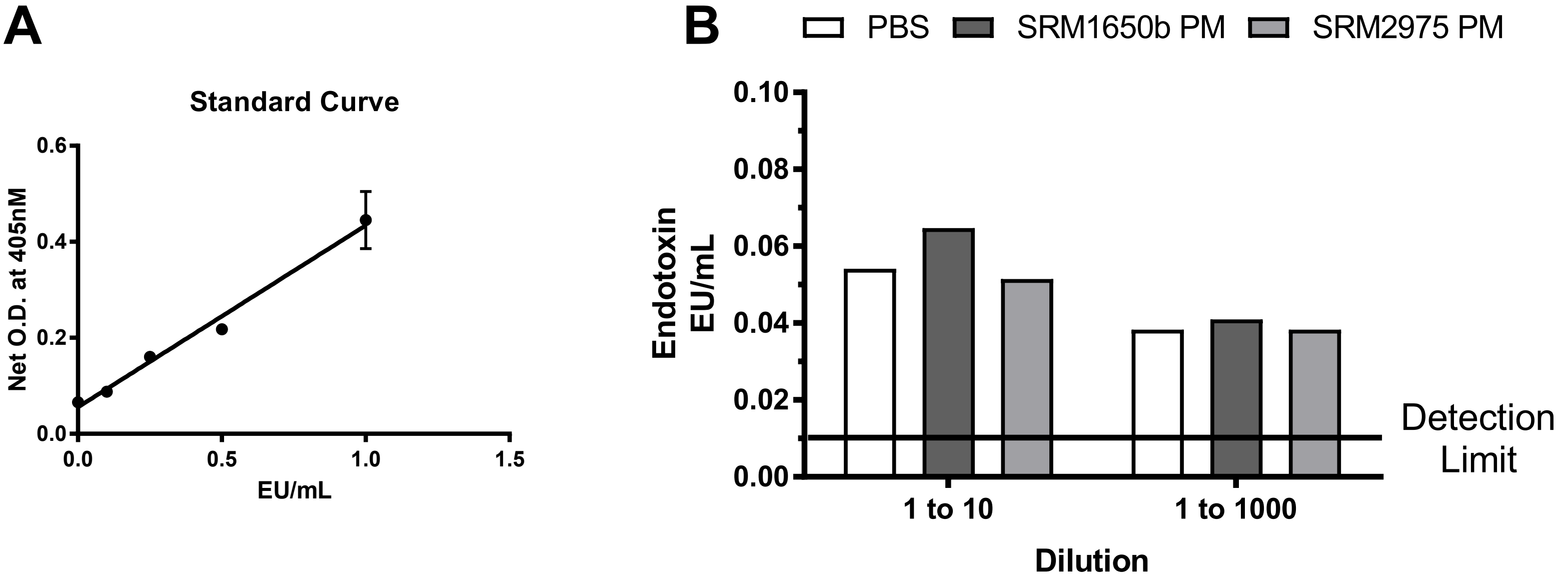
A
Standard Curve
Net O.D. at 405nM
0.6
0.4
0.2
0.0
0.0
0.5
1.0
1.5
EU/mL
B
PBS
SRM1650b PM
SRM2975 PM
Endotoxin EU/mL
0.10
0.08
0.06
0.04
0.02
0.00
Detection Limit
1 to 10
1 to 1000
Dilution

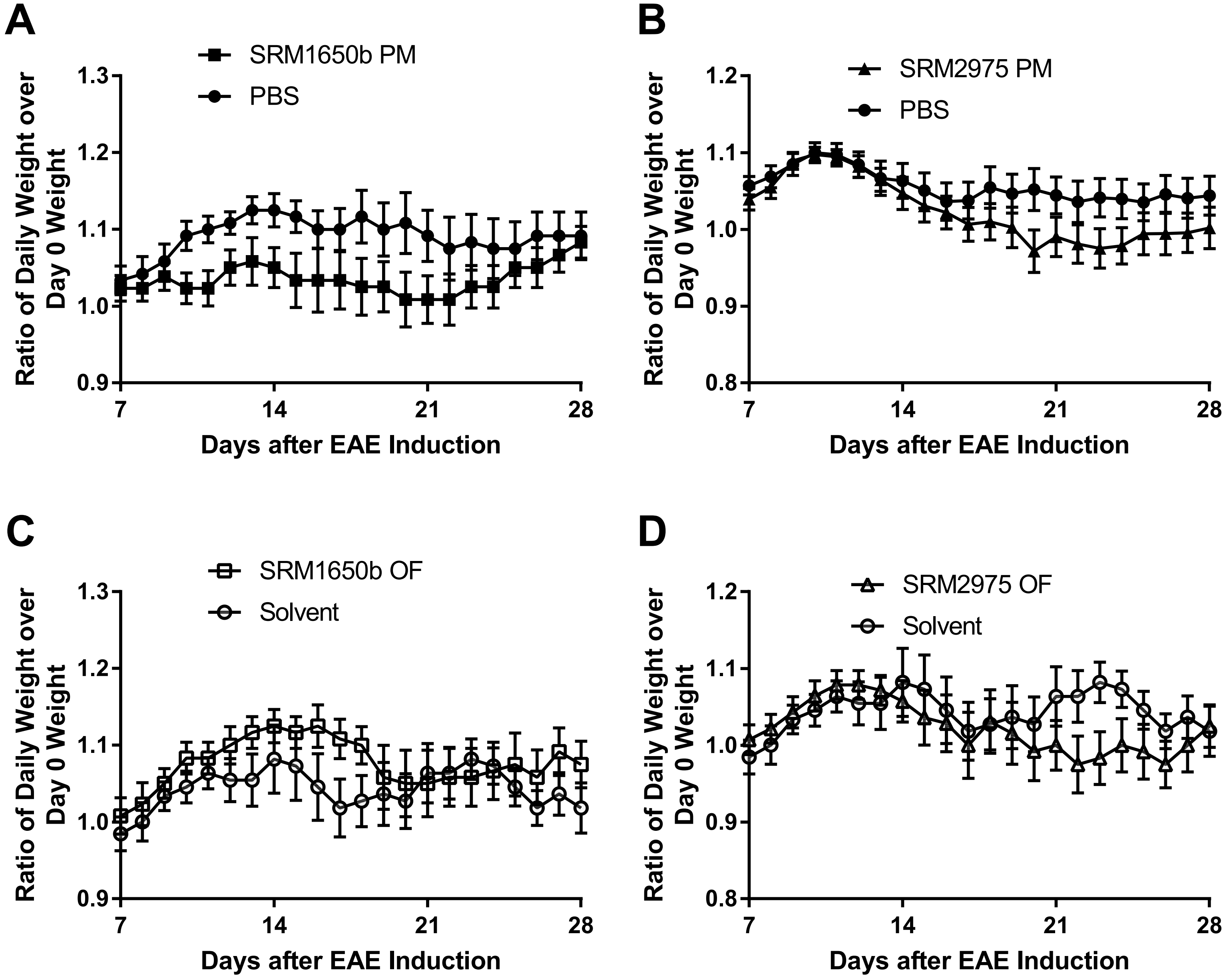

A
SRM1650b PM
PBS
Ratio of Daily Weight over Day 0 Weight
Days after EAE Induction
1.3
1.2
1.1
1.0
0.9
7
14
21
28
B
SRM2975 PM
PBS
Ratio of Daily Weight over Day 0 Weight
Days after EAE Induction
1.2
1.1
1.0
0.9
0.8
7
14
21
28
C
SRM1650b OF
Solvent
Ratio of Daily Weight over Day 0 Weight
Days after EAE Induction
1.3
1.2
1.1
1.0
0.9
7
14
21
28
D
SRM2975 OF
Solvent
Ratio of Daily Weight over Day 0 Weight
Days after EAE Induction
1.2
1.1
1.0
0.9
0.8
7
14
21
28

PAHs	SRM1650b			SRM2975		
	ng PAH/mL extracted	ng PAH/ 10µg/mL dose exposure	ng/PAH per cell at 10µg/mL dose exposure	ng PAH/mL extracted	ng PAH/ 10µg/mL dose exposure	ng/PAH per cell at 10µg/mL dose exposure
Phenanthrene	10943	0.76091	5.0727E-06	2529.13	1.7923	1.1529E-05
Anthracene	689.50	0.04794	3.1963E-07	0	0	0
Fluoranthene	11568	0.80437	5.3625E-06	5801.2	3.9666	2.6444E-05
Pyrene	10613	0.73795	4.9196E-06	0	0	0
Benz[a]anthracene	2451.2	0.17044	1.1363E-06	0	0	0
Chrysene	9680.4	0.67313	4.4875E-06	1487.5	1.0171	6.7804E-06
Benzo[b]fluoranthene	1913.9	0.13308	8.8721E-07	1956.6	1.3379	8.9192E-06
Benzo[k]fluoranthene	1153.9	0.080233	5.3489E-07	0	0	0
Benzo[e]pyrene	1522.0	0.10583	7.0554E-07	164.27	0.11232	7.4881E-07
Benzo[a]pyrene	317.85	0.022102	1.4735E-07	0	0	0
Perylene	0	0	0	0	0	0
Indeno[1,2,3-cd] pyrene	1391.48	0.096756	6.4504E-07	249.72	0.17075	1.1383E-06
Benzo[g,h,i]perylene	1550.83	0.10784	7.1891E-07	0	0	0
Dibenz[a,h]anthracene	0	0	0	0	0	0
Picene	0	0	0	0	0	0

Table S1: PAH concentrations in OF extracts. PAH concentrations of 15 EPA PAHs of concern for SRM1650b and SRM2975 OF. The ng of each PAH that was extracted using the DCM/DMSO solvent extraction was measured. The PAH concentrations are shown in ng of individual PAHs present in the highest 10µg/mL OC exposure of the OF. Additionally, ng of PAH per cell in the highest 10µg/mL OC exposure was also calculated.

PAHs	SRM1650b			SRM2975		
	ng PAH	ng PAH/ 10µg/mL OC exposure	ng PAH/ cell at 10µg/mL OC exposure	ng PAH	ng PAH/ 10µg/mL OC exposure	ng PAH/ cell at 10µg/mL OC exposure
Phenanthrene	10.948	0.72989	4.8659E-06	12.641	1.6855	1.1237E-05
Anthracene	0.68984	0.045990	3.0660E-07	0	0	0
Fluoranthene	11.573	0.77158	5.1438E-06	28.771	3.8362	2.5575E-05
Pyrene	10.618	0.70786	4.7191E-06	0	0	0
Benz[a]anthracene	2.4524	0.16350	1.0900E-06	0	0	0
Chrysene	9.6853	0.64568	4.3046E-06	7.3772	0.9836	6.5575E-06
Benzo[b]fluoranthene	1.9148	0.12766	8.5104E-07	9.7041	1.2939	8.6259E-06
Benzo[k]fluoranthene	1.1544	0.07696	5.1308E-07	0	0	0
Benzo[e]pyrene	1.5227	0.10152	6.7678E-07	0.8147	0.1086	7.2419E-07
Benzo[a]pyrene	0.31801	0.02120	1.4134E-07	0	0	0
Perylene	0	0	0	0	0	0
Indeno[1,2,3-cd] pyrene	1.39218	0.092812	6.1875E-07	1.2385	0.1651	1.1009E-06
Benzo[g,h,i]perylene	1.5516	0.10344	6.8960E-07	0	0	0
Dibenz[a,h]anthracene	0	0	0	0	0	0
Picene	0	0	0	0	0	0

Table S2: PAH mixture PAH concentrations. PAH concentrations of 15 EPA PAHs of concern for SRM1650b and SRM2975. The PAH concentrations were measured in ug/mL of PAH extracted as well as ng of individual PAHs present in the highest 10µg/mL OC exposure. Additionally, ng of PAH per cell at the highest 10µg/mL dose was calculated.

	SRM1650b			SRM2975		
**Dose (µg/mL PM)**	**pg PM/cell**	**µg OC/mL**	**pg OC/cell**	**pg PM/cell**	**µg OC/mL**	**pg OC/cell**
40	0.17	9.0	0.038	0.17	2.3	9.9E-03
20	0.043	4.5	9.6E-03	0.043	1.2	2.5E-03
12.5	0.017	2.8	3.8E-03	0.017	0.73	9.7E-04
3.125	0.0010	0.70	2.3E-04	0.0010	0.18	6.0E-06
0.78	0.00013	0.18	2.9E-05	0.00013	0.050	7.5E-06

Table S3: PM *in vitro* dose conversion. This table converts the PM doses based on mass of PM to mass of organic carbon. Additionally, mass of PM and mass of OC per cell are calculated.

	SRM1650b			SRM2975		
**Dose (ug/mL OC)**	**pg OC/cell**	**µg PM/mL**	**pg PM/cell**	**pg OC/cell**	**µg PM/mL**	**pg PM/cell**
10	4.667E-03	44.44	0.02074	0.04533	172.4	0.7816
5	2.333E-03	22.22	0.0137	0.02267	86.21	0.3908
1	4.667E-04	4.444	2.074E-03	4.533E-03	17.24	0.07816
0.1	4.667E-05	0.4444	2.074E-04	4.533E-04	1.724	7.816E-03
0.01	4.667E-06	0.04444	2.074E-05	4.533E-05	0.1724	7.816E-04
0.001	4.667E-07	0.004444	2.074E-06	4.533E-06	0.01724	7.816E-05
0.0001	4.667E-08	0.0004444	2.074E-07	4.533E-07	0.001724	7.816E-06
0.00001	4.667E-09	0.00004444	2.074E-08	4.533E-08	0.0001724	7.816E-07

Table S4: OF *in vitro* dose conversion. This table converts the OF and PAH mixture doses based on organic carbon to a measure based on mass of PM. Additionally, mass of OC and mass of PM per cell are calculated.
